# Inferring Stop-Locations from WiFi

**DOI:** 10.1371/journal.pone.0149105

**Published:** 2016-02-22

**Authors:** David Kofoed Wind, Piotr Sapiezynski, Magdalena Anna Furman, Sune Lehmann

**Affiliations:** DTU Compute, Technical University of Denmark, Copenhagen, Denmark; Beijing University of Posts and Telecommunications, CHINA

## Abstract

Human mobility patterns are inherently complex. In terms of understanding these patterns, the process of converting raw data into series of *stop-locations* and *transitions* is an important first step which greatly reduces the volume of data, thus simplifying the subsequent analyses. Previous research into the mobility of individuals has focused on inferring ‘stop locations’ (places of stationarity) from GPS or CDR data, or on detection of state (static/active). In this paper we bridge the gap between the two approaches: we introduce methods for detecting both mobility state and stop-locations. In addition, our methods are based exclusively on WiFi data. We study two months of WiFi data collected every two minutes by a smartphone, and infer stop-locations in the form of labelled time-intervals. For this purpose, we investigate two algorithms, both of which scale to large datasets: a greedy approach to select the most important routers and one which uses a density-based clustering algorithm to detect router fingerprints. We validate our results using participants’ GPS data as well as ground truth data collected during a two month period.

## Introduction

With the growing availability of datasets describing human behavior, it has become increasingly feasible to study mobility of individuals and entire social systems [1]. Large-scale records of human mobility can be used to, for example, model spreading of epidemics [2, 3], infer and analyze social networks [4, 5], or to quantify and understand fundamental properties of our behavior, such as predictability [6, 7].

Early mobility research focused primarily on call detail records (CDR) data made available by telecom operators [1]. Such datasets cover large populations—the operators’ entire customer bases—but contain biases in terms of sampling and spatial resolution. These biases might result in an underestimation of individuals’ mobility [8]. On the other hand, the use of GPS data enables a high spatial resolution that allows for accurate estimation of mobility, especially with respect to discovery of stay points and places of interest [9–11]. GPS information is, however, rarely available for populations of comparable size to mobile phone datasets due to, for example, high battery impact [12] and the perceived impact on privacy of such data [13].

Using WiFi as a data source for detecting and classifying mobility is a well-studied research problem. It is possible to calculate the position of a device with accuracy of under 1.5 meters using trilateration [14], but this strategy has only been shown to work indoors and requires an expensive training phase. One can also classify the mobility state by investigating variance of Received Signal Strength Indication (RSSI), but such approaches require temporal resolution of the data as high as one sample per two seconds [15, 16] and robustness to lower- or variable sampling rates has not yet been demonstrated.

Here we show how to identify stop-locations using WiFi data exclusively. There are multiple motivations for using WiFi data in place of GPS data: First of all, WiFi information is potentially available for large populations. For example, at the time of writing (Q1 2015), 17 out of 20 top free games on Android Play Store required access to WiFi information, while none of them required access to GPS data. Moreover, because of frequent WiFi scans scheduled by the Android operating system (by default even when the user disables WiFi), the WiFi information can be obtained by applications without additional cost to the battery [17].

Secondly, related to the study of human behavior, sequences of latitude and longitude coordinates are not how human beings process location. We argue that a sequence of stop locations is a more natural representation of a day’s activities. An example of a set of stop-location is given below.

17:33 – 07:32: Home

07:40 – 08:07: Coffee shop

08:18 – 16:10: Work

With data represented as labelled intervals, we are able phrase research questions more directly, for example ‘How does the time spent at work relate to *x*’, where the time spent at work can now be found by adding up the lengths of the intervals labelled ‘work’. Thirdly, in contrast to the GPS representation where mobility is represented as a sequence of pairs of rational numbers (coordinates on a sphere), an individual’s stop-locations constitute a finite alphabet, which we can analyze using, for example, the tools of information theory. Thus, the stop-location representation greatly reduces the dimensionality and sheer volume of data.

In the literature different methods have been developed to extract such personal diaries from data sources such as GPS [10]. Here, we define a stop-location as a location in which a subject is stationary—defined by a start time, an end time and optionally a label for the location. The intervals between stop-locations are denoted *trips*.

When considering human mobility and especially when inferring stop-locations of people, there is an inherent problem of scale [18–21]. When sitting at your office desk, there are multiple correct stop-locations to report: your chair, your office, your building, your city, your country. Which of these scales to report, depends on the application. Since WiFi data is very local (a typical router has a range of up to around 100 meters), the stop-locations that we can infer based on WiFi are on a scale corresponding to buildings.

### Data

The ground truth data was collected using a smartphone (LG Nexus 4 running Android 4.4.3) with software that periodically scans and records scans for WiFi (visible access points), Bluetooth (visible Bluetooth devices) and GPS (location coordinates) [22, 23]. The dataset was collected by a single individual and runs over a period of 60 days between September 9th, 2014 and November 8th, 2014, and contains 41441 WiFi scans (approximately one every second minute), 5982 unique WiFi devices. In total 25161 GPS samples were collected (about one every 3–4 minutes). Over the data collection period 137 stops were recorded. In addition to the automatic recording of WiFi and GPS, the subject manually recorded which state she was in (bike, bus, car, run, stand, train or walk) at all times. It should be noted that the stationary (‘stand’) entries were not labelled to indicate specific location. A part of this diary is shown below:

09-09-2014 16:00 stand

09-09-2014 17:22 walk

09-09-2014 17:23 bike

09-09-2014 17:35 stand

09-09-2014 17:36 walk

09-09-2014 17:37 stand

09-09-2014 17:38 train

**Fig 1:**
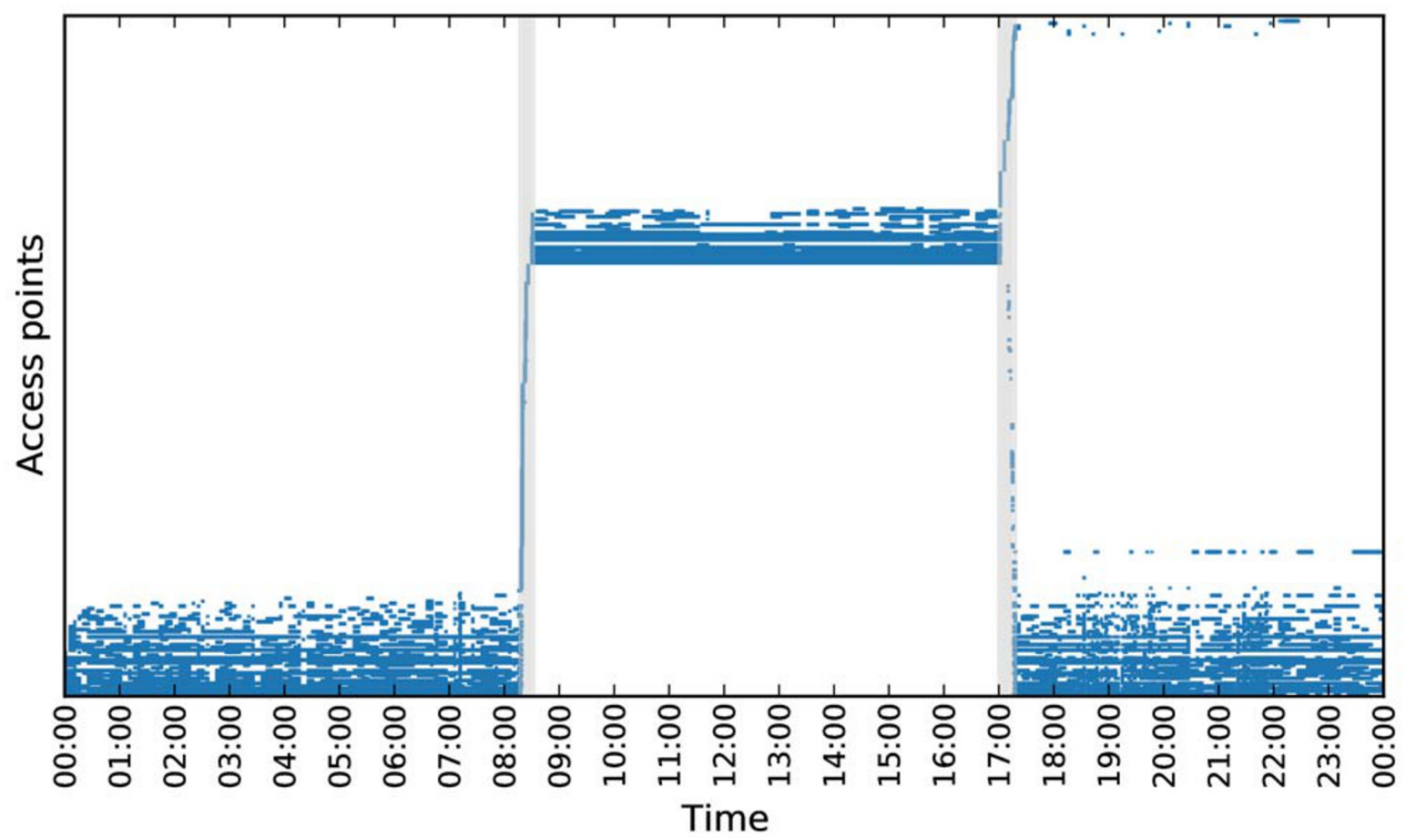
A visualization of a single day of WiFi scans as a matrix. Each row in the matrix corresponds to an access point and each column to a point in time. A cell in the matrix is filled if the access point was observed at that specific time. Columns which correspond to transitions between stop-locations (labelled according to ground truth) are colored in gray. The rows are ordered by the first time an access point is observed.

One day of the collected WiFi data is visualized in Fig 1. We use this diary of mobility as ground truth to evaluate the accuracy of the algorithms for inferring stop-locations based on the automatically collected WiFi data. Data collection, anonymization, and storage were approved by the Danish Data Protection Agency, and comply with both local and EU regulations.

### Structure of this paper

The remainder of the paper is organized as follows. In Section 1 we describe methods for inferring stop-locations based on mobile sensing data. We start by discussing a recent algorithm based on GPS data [10], which we use as a baseline for our two novel approaches. We then discuss WiFi-algorithm 1 (Greedy Router Selection), which uses the most prevalent single routers and treats them as locations. WiFi-algorithm 2 (Density Based Clustering of Time Samples) uses clustered routers as locations. In Section 2 we use two different evaluation schemes to compare the stop-locations found by the different methods. Finally, in Section 3 we discuss the advantages and shortcomings of the different methods, address potential issues of our analysis and propose future work.

## 1 Methods

### Distance Grouping and Density Based Clustering of GPS Samples

In order to evaluate the usefulness of employing WiFi in order to infer stop-locations, we compare our results to stop-locations obtained using GPS, using a state-of-the-art method [10], which employs a combination of distance grouping and Density Based SCAN (DBSCAN) [24]. The distance grouping algorithm is based on the idea that a stop corresponds to a temporal sequence of locations within a maximal distance *d*_max_ from each other. Locations are examined sequentially by non-decreasing timestamp. Each stop initially contains only a single location loc_*i*_, and each subsequent location loc_*i*+*k*_ is added to the stop while distance(loc_*i*+*k*_, loc_*i*_) < *d*_max_. Then the process is restarted from loc_*i*+*k*+1_. After the distance grouping is complete, we are left with a number of groups of locations, each corresponding to a stop. Within each group the geometric median (the point minimizing the sum of distances to the points in the group) is identified and finally DBSCAN is run on the set of medians, yielding a number of clusters—each corresponding to a place of interest. The DBSCAN algorithm requires specification of two parameters *ε* and *M*. The *ε*-parameter dictates that if two points are within distance *ε* from each other, they belong to the same cluster. The *M*-parameter specifies the minimum number of points in a cluster. In Ref. [10], *d*_max_ = 60m and DBSCAN has parameters *ε* = 60m and *M* = 1. The distance metric is the haversine metric.

### Greedy Router Selection

The greedy approach was to router selection was originally proposed as a method for reducing the WiFi scan data volume in order to describe the mobility using as few routers as possible [17]. Here, we show that routers selected using this method correspond to stop-locations.

#### Method

We quantize the timestamps of WiFi samples’ into 5 minute time bins, corresponding to the sampling rate of WiFi in the data collector app (more samples may be available due to passive scanning in Android). Next, we sort the list of all routers by the number of unique time bins in which they appear. We then select the most most frequently occurring router and define its set of time bins as *covered time bins*. The next step is to descend through the sorted list of routers and find the router for which the union of covered time bins with its respective time bins is has the most elements, while discarding the routers with majority of time bins already covered. This step identifies the router, for which the increase in covered time bins is the largest. The new union is now defined as *covered time bins* and the search is restarted, from top of the list. The algorithm stops where no routers can be found to extend the set of covered time bins by at least *ΔN* (we use *ΔN* = 1 for simplicity). This results in a list of important routers which is much smaller than the set of all routers (typically, 20 routers are enough to describe the location of a person 90% of time [17]).

#### Post-processing

Upon extracting the important routers, we label each scan in which they appear as a ‘stop location: routerid’. Scan results which do not contain any of the important routers are labelled as ‘moving’ state. In order to achieve results comparable with the method presented in [10], we discard all stop locations with duration lower than 15 minutes. We also discard all moving states of duration lower than 15 minutes if their adjacent stop locations correspond to the same important router.

### Density Based Clustering of Time Samples

As an alternative to the—potentially non-optimal—greedy method of using single routers as stop-locations, we propose a method which uses multiple routers as a ‘finger print’ of a stop-locations below.

#### Data

From the WiFi samples, we construct a data matrix *X* with each row corresponding to an observed router, and each column corresponding to time stamp for which we have a WiFi-sample. The element *X*_*r*,*t*_ is set equal to 1 if we observe the router *r* in the sample at time *t* and 0 otherwise. Since each WiFi-sample only contains a small portion of the total set of routers in the data set, the columns of this matrix are very sparse (see Fig 1). The rows are not necessarily sparse, since some routers are observed a large percentage of the time.

#### Pre-processing

Before inferring the stop-locations for the user, we pre-process the matrix. First we bin the data by introducing a time-grid with 5-minute intervals—once again corresponding to wifi sampling rate—and merging WiFi-samples occurring within the same 5-minute interval. In this column merge-step, pairs of subsequent WiFi-observations are combined using a union of the corresponding binary columns (corresponding to observing all routers from both samples at the same time).

Second we merge routers (rows) which are a subset of another router to remove a number of routers which insignificant. As part of the row merge-step the same time we introduce a weighting of the importance of the routers, where each router *r* starts off with an initial weight *w*(*r*) of 1. Now, given *r*_*a*_ and *r*_*b*_ where observations of *r*_*b*_ are a strict subset of *r*_*a*_ observations, then we remove the row corresponding to *r*_*b*_, and update the weight of *r*_*a*_ to $w\left(r_{a}\right)\to w\left(r_{a}\right)+\frac{|r_{b}|}{|r_{a}|}$, where |*r*| is the number of observations of router *r* in the data set. In the cases where a router *r*_*x*_ is a subset of multiple routers *R* = *r*_1_, …, *r*_*n*_, we choose a random router *r*_*y*_ ∈ *R* and merge *r*_*x*_ into *r*_*y*_.

**Fig 2:**
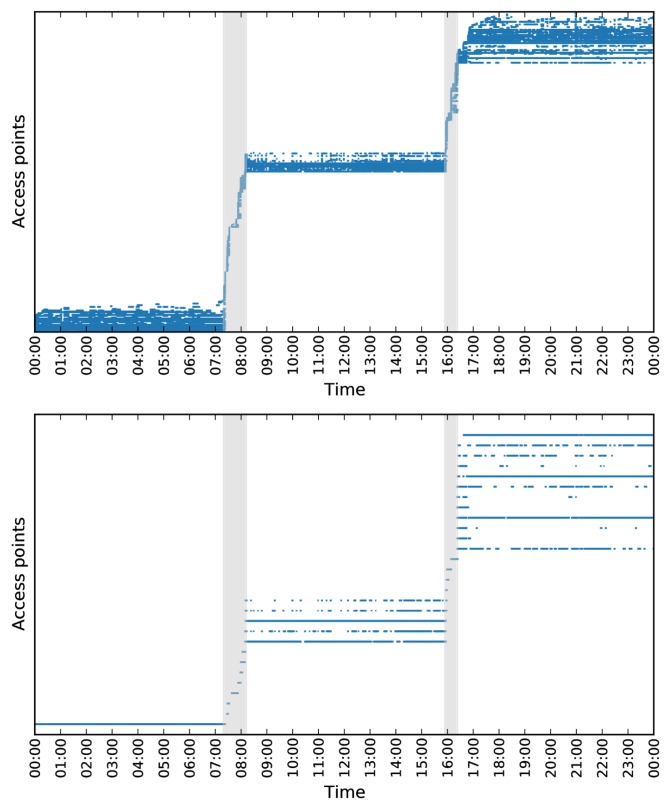
Visualization of merge step for density based clustering. By merging two routers when one of them is a complete subset of the other, we reduce the number of routers in the data set. Here, merging is illustrated for a single day of data. The resulting reduction is from 357 to 29 routers. Note that the first stop-location has been reduced to a single router.

These two merge-steps result in a sparse matrix *X*′, where no rows are subsets of each other, and a vector of weights *W*. In Fig 2 a part of the data matrix *X* is shown before the merging of routers and a part of *X*′ after the merging of routers.

#### Clustering

To identify stop-locations, we assign the columns of *X*′ clusters using the DBSCAN (Density Based SCAN) algorithm [24]. As above, we must determine the value of DBSCAN’s two parameters: *ε* and *M* which are dependent on the problem. Further, we need to select a suitable distance measure for comparing pairs of WiFi-samples.

The Jaccard-distance of two binary vectors *x* and *y* is defined as:

$$\begin{array}{c} J\left(x,y\right)=1-\sum_{i=0}^{N}(\frac{I\left(x_{i}\right)I\left(y_{i}\right)}{I\left(x_{i}\right)+I\left(y_{i}\right)-I\left(x_{i}\right)I\left(y_{i}\right)}) \end{array}$$ (1)

where *I*_*v*_*i*__ is an indicator function taking the value 1 if and only if the *i*-th element of the vector *v* is 1. We use a weighted version of the Jaccard-distance defined in Eq (2):

$$\begin{array}{c} J_{W}\left(x,y\right)=1-\sum_{i=0}^{N}(\frac{w_{i}I\left(x_{i}\right)I\left(y_{i}\right)}{I\left(x_{i}\right)+I\left(y_{i}\right)-w_{i}I\left(x_{i}\right)I\left(y_{i}\right)}) \end{array}$$ (2)

In order to avoid cases when sporadic noise result in thew clusters, we choose *M* to be larger than 1, but keep the value as low as possible (in this case *M* = 2); this allows for stop-locations which were visited only once in the data set. The parameter *ε* = 0.325 was chosen as to match stop-locations on the building-scale.

If we want to run this method live on incoming data (in an online fashion), we can easily update the stop location and regularly recalculate which routers should be merged. When we observe a new time-sample *x*_*t*_, we it to a cluster by letting *x*_*t*_ belong to a cluster *C* when the Jaccard-distance between *x*_*t*_ and some point in *C* is less than *ε*. Due to the sparsity of the samples (columns of *X*) and the nature of the data (that most pairs of routers never appear together and some almost always do), we can efficiently compute which cluster a new sample belongs to by maintaining a data-structure for finding close points to a new point.

**Fig 3:**
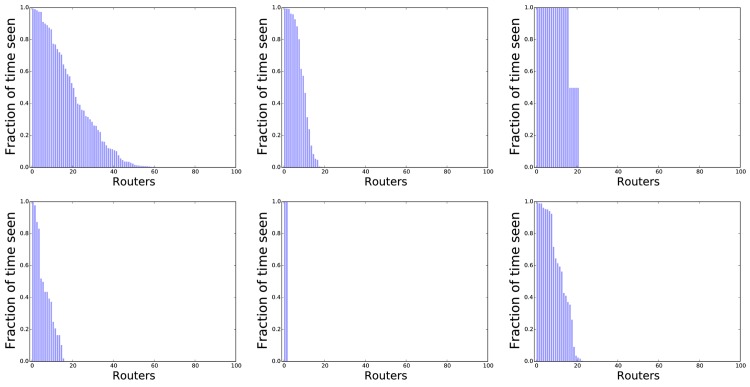
Six examples of the distribution of routers in a cluster. Each plot corresponds to a single-cluster obtained from DBSCAN. In a plot, each bar (a maximum of 100 bars is shown) corresponds to an access-point, and its height corresponds to the proportion (0 to 1) of the samples in the cluster where the router was present. In most of the clusters, 1–10 routers are all present 100% of the time.

Using this method, each inferred cluster can be viewed as a ‘fingerprint’ specifying the routers that are typically present at the corresponding stop-location. In Fig 3 we have visualized the distribution of router-presence at a few representative stop-locations. Most clusters contain more than a single router, indicating that the method achieves robustness to a single router disappearing—and many clusters have 1–10 routers appearing 100% of the time.

#### Post-processing

After clustering the time-samples, we perform the following post-processing step: A sequence of clusters *A*, *B*, *A*, is merged to a single occurrence of cluster *A*, if the stop in cluster *B* is shorter than 15 minutes. We also merge two consecutive occurrences of the same cluster if the gap between them is smaller than 15 minutes. These post-processing steps are performed in order to achieve results comparable with the baseline method presented in [10].

## 2 Evaluation and Results

Below we compare the stop-locations inferred by each of the three different methods presented above to the ground truth stop-locations. The problem of inferring stop-locations introduces two challenges. One challenge is to detect *when* a subject is stationary (which is equivalent to detecting when a subject is transitioning between stop-locations) and another is to infer in *which* stop-location the subject is stationary. Therefore, we perform two different tests, one evaluating at how well each method can predict the start and stop-times of each stop recorded in the ground truth, and one investigating how well the different methods are able to infer stop-locations, which match the true stops in regards to their geographical location.

### Overlap of stop-locations

**Fig 4:**
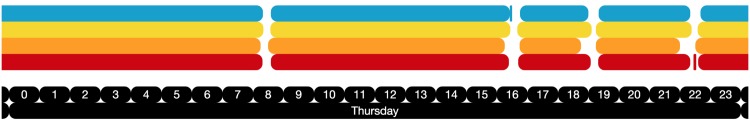
An example of how the stop-locations inferred by the different methods compare to the ground truth stop-locations. The bottom timeline (red) is the stop-locations as reported by the ground truth. The first time line (blue) is the one obtained using DBSCAN on WiFi. The second time line (yellow) is the one obtained using the greedy router selection, and the third timeline (orange) is the one obtained using GPS data.

To quantify the estimation of start- and stop times for the different algorithms, we measure the overlap between stop-locations found by each method and the ones given in the ground truth. A visualization of the stop-locations found by the different methods is displayed in Fig 4.

Because the ground truth data does not contain labels for the stop-locations, we consider the problem to be a binary classification problem, where the task is to predict whether or not the subject is stationary in a given time bin. We split the time-axis into bins with length 1 minute, and count in how many bins each method agrees with the ground truth, and in how many it disagrees. If the start and stop times for the inferred stop-locations are different than the ground truth, this will result in misclassifications. We compare the stop-locations found using GPS-traces, the ones found using greedy router selection, the ones found using DBSCAN on the WiFi-data and a baseline metric always predicting that the subject is in a stop-location (since approximately 96% of the time is spent in a stop-location).

We use 5 different metrics to compare the methods:

$$\begin{array}{ccc} \text{Classification}\;\text{error} & : & \;\frac{FP+FN}{P+N}\\ \text{Precision} & : & \;\frac{TP}{TP+FP}\\ \text{Recall} & : & \;\frac{TP}{TP+FN}\\ F_{1}\text{-score} & : & \;\frac{2TP}{2TP+FP+FN}\\ \text{MCC} & : & \;\frac{TP\times TN-FP\times FN}{\sqrt{\left(TP+FP)(TP+FN)(TN+FP)(TN+FN\right)}} \end{array}$$

where *P* is the number of times the subject was in a stop location, *N* is the number of times the subject was not in a stop location, *TP* is the number of times the model correctly predicts that the subject is in a stop-location, *TN* is the number of times the model correctly predicts that the subject is not in a stop-location, *FP* is the number of times the model falsely predicts that the subject is in a stop-location, and *FN* is the number of times the model falsely predicts that the subject is not in a stop-location. Matthews Correlation Coefficient (MCC) is a measure of the quality of a binary classification; it is generally regarded as a balanced measure which can be used for problems with large class imbalance (which is the case here, since people are mostly stationary). Even with a very high fraction of time-bins where the subject is stationary, a simple model always predicting stationarity will receive a MCC of 0.

The results are summarized in Table 1. The greedy router selection achieves the highest classification rate of 98.1%, where the GPS-based method achieves a rate of 94% and the always-one baseline gets an accuracy of 96%. In the *F*_1_-metric, the two WiFi-based methods achieve a score of 0.990, the GPS-based a (lower) score of 0.969 and the always-one baseline a score of 0.979. The WiFi-based DBSCAN gets a Matthew’s Correlation Coefficient of 0.737, the greedy router selection scores 0.723, the GPS-based method gets a 0.497 and the always-one baseline scores a MMC of 0.

**Table 1:** The results when evaluating the different methods ability to find stop-locations overlapping with the ground truth. We report 5 different error measures for each method. DBSCAN-method on WiFi data achieves the best result for Matthew’s Correlation Coefficient. One reason that the GPS-based method yields the highest precision is that mobile routers are inferred as stop-locations for the WiFi based methods, but are not reported as such in the ground truth.

	GPS	DBSCAN	Top router	Always 1
**Classification error**	0.060	0.020	**0.019**	0.040
**Precision**	**0.989**	0.988	0.985	0.960
**Recall**	0.950	0.992	0.995	**1.000**
**F**_1_	0.969	**0.990**	**0.990**	0.979
**MCC**	0.497	**0.737**	0.723	0.000

### Median distance between stop-locations

We now study how well each method is able to infer *in which* stop-location the subject is stationary. Because our ground truth data does not include labels of the recorded stops, we are not able to easily quantify whether the stops found by the methods correspond to physical locations of interest. Using the GPS-samples collected along with the WiFi data, we therefore evaluate if the clusters found by the different methods are geographically close to the stops recorded in the ground truth. In order to quantify how well the stop-locations inferred from the data correspond to the true stop-locations coordinates, we compare the geographical median of each inferred stop-location to the geographical median of GPS-samples in the ground truth.

For each recorded stop (*g*_s*tart*_, *g*_e*nd*_) in the ground truth data, we determine if the method predicts a stop in cluster *c* which is at least 70% overlapping with (*g*_s*tart*_, *g*_e*nd*_). We have to select some threshold for how big an overlap two stops need to have before we compare them due to the inherent problem of scale in detecting stop-locations. The threshold of 70% can be chosen anywhere between 55% and 85% giving similar results.

**Fig 5:**
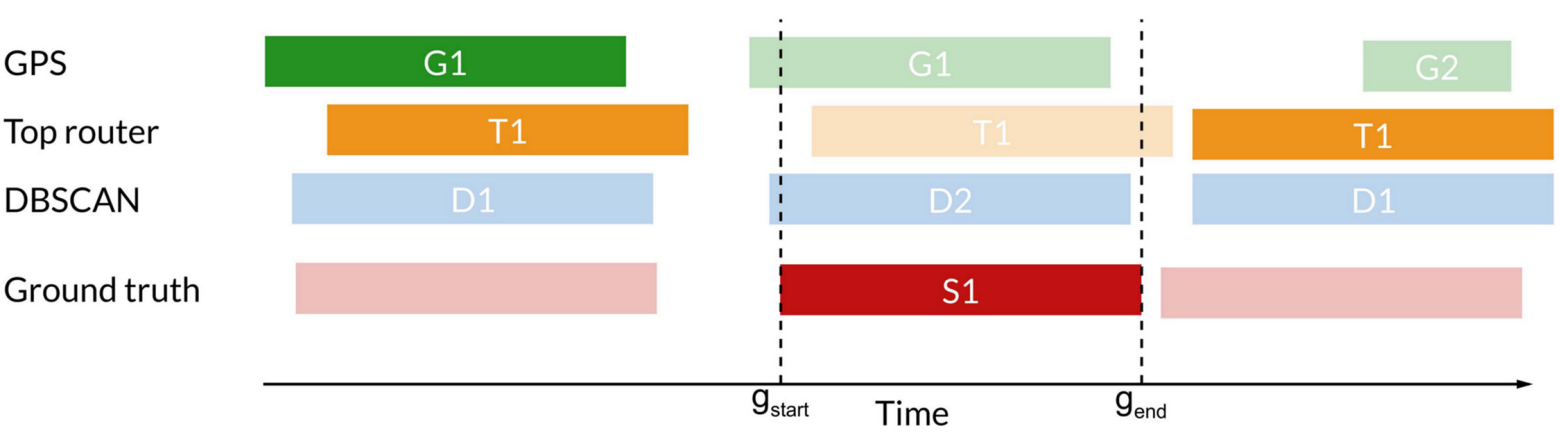
During the ground truth stop between time *g*_start_ and *g*_end_ (labeled *S*1), the GPS-method reports cluster *G*1, the Top-router method reports cluster *T*1 and the DBSCAN-method reports cluster *D*2. Now we want to compare the geographical median of *S*1 to clusters *G*1, *T*1 and *D*2. We do this by—for each method—computing the distance between the geographical median of the gps-samples collected during *S*1 and the geographical median of the gps-samples collected during for example *G*1, excluding the ones collected during *S*1 (to avoid overfitting). In the figure, this is depicted by comparing samples from *S*1 to samples from the non-grayed-out *G*1.

If this is the case, then we compare the geographical median of the GPS-samples collected within (*g*_s*tart*_, *g*_e*nd*_) to all GPS-samples happening while the method predicts cluster *c* except for those occurring in (*g*_s*tart*_, *g*_e*nd*_) (to avoid using the same GPS-samples data for computing the two medians). See Fig 5 for a visualization of this.

**Fig 6:**
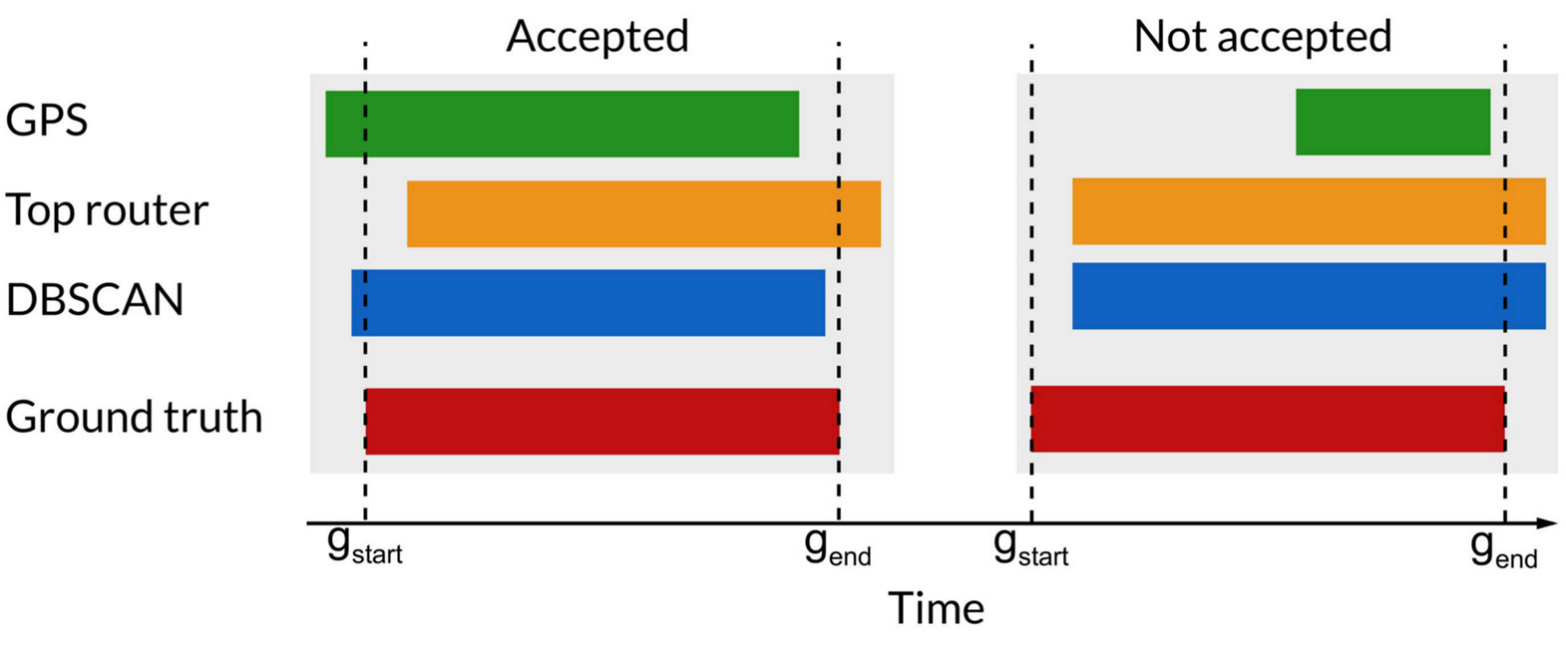
We only make the comparison of medians for the ground truth stops where all methods report stops with at least 70% overlap. In this figure the first example (on the left) is used for comparison whereas the second (on the right) is not since the GPS method does not report a sufficiently overlapping stop. *g*_start_ and *g*_end_ refers to the starting and stopping times of the ground truth stop-location.

**Fig 7:**
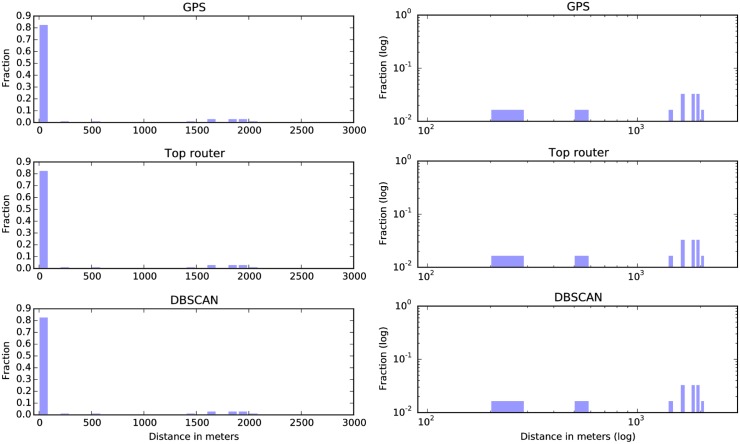
The distribution of distances between the true stop median position and the median position reported by the three methods. The histograms in the right column are log-log versions of the figures in the left column. As seen, most error-distances are less than 100 meters, but a few large errors of around 2000 meters are reported by all methods.

We perform this comparison for all reported stop-locations in the ground truth where every method (GPS, DBSCAN on WiFi and Greedy Router Selection) reports a stop-location with 70% overlap to the ground truth stop (see Fig 6 for a visualization of this). The distribution of the distance between the true stop median position and the median position reported by the three methods is shown in Fig 7.

For the three methods, the median of the distance between the median position of the stops found using GPS-traces and the true stop position is 28.86 meters. For DBSCAN on WiFi, the median error is 29.17 meters and for the Greedy router selection, the median error is 29.26 meters. This metric penalizes methods which end up with clusters corresponding to two or more different geographical stop-locations. The reason is that in this case, the geographical coordinates for the center of the cluster (which is the geographical median) will be far off from at least one of the ground truth stops.

## 3 Discussion

Above, we have analyzed the feasibility of inferring human mobility in the form of stop-locations using WiFi data. The analysis is based on two months of smartphone based WiFi data. We proposed two different approaches to inferring stop-locations from WiFi data, one based on greedily selecting routers as stop-locations and one using router signature finger printing with DBSCAN. Each method was evaluated using two evaluation schemes and compared to a baseline method utilizing GPS-data for stop-location inference. The evaluation schemes measured a) how well the start and stop-time of the stop-locations match the ground truth, and b) how well the geographical medians of the inferred stops correspond to the ground truth data.

In the evaluation of start and stop-times, the WiFi based methods outperform the GPS-based method, primarily because of the higher sampling rate for WiFi. In the evaluation of the geographical precision of the stops, all the methods report similar errors. In general, our results demonstrate that it is feasible to infer stop-locations using WiFi. That two different approaches to inferring stop-locations with WiFi (greedy router selection and DBSCAN) both work, indicates that WiFi is a robust data source for this application.

The greedy router selection approach is straightforward to implement, computationally efficient and produces results which can be easily interpreted. However, due to the lack of knowledge of other routers surrounding the selected access points, the results are not robust. Whenever one of the important routers is replaced by another device in its location, it is not possible recognize and merge the new label with the previous one. Similarly, when one of the important routers is moved to a new physical location, it is not possible to *not* merge the two places.

**Fig 8:**
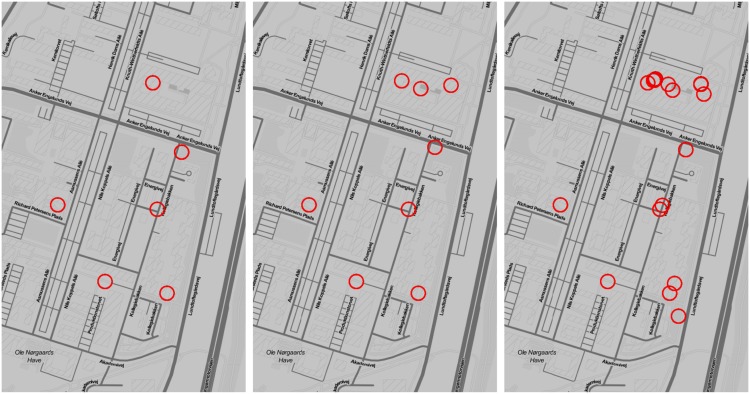
The three approaches produce a different number of points of interest. Density based clustering of GPS data (left) produces the lowest number of stop locations, followed by greedy selection of routers (middle), and DBSCAN (right). All the stops from GPS are reflected using WiFi data, but WiFi based methods identify locations with a higher spatial resolution.

None of the methods described in this paper require a specification of the number of stop-locations to find. This is an advantage because the problem of scale makes it impossible to give an objectively correct estimation of this. The three different methods find very different number of clusters (see Fig 8 for an example). The GPS-based method infers 16 distinct clusters, the greedy single-router based method infers 35 distinct clusters, and the DBSCAN-based WiFi method infers 69 distinct clusters. Adding to the complexity of the problem, the number of clusters found by the different methods is strongly dependent on the parameters of each method. For the GPS-based method, the parameters are *d*_max_ and the two parameters *ε* and *M* for DBSCAN. For the greedy router selection the parameter is *ΔN*. For the DBSCAN-based WiFi method, the parameters are *ε* and *M* for DBSCAN. Additionally all methods have variability in their pre- and post processing steps, for example the bin-size when time-binning and removal of *short* stop-locations.

**Fig 9:**
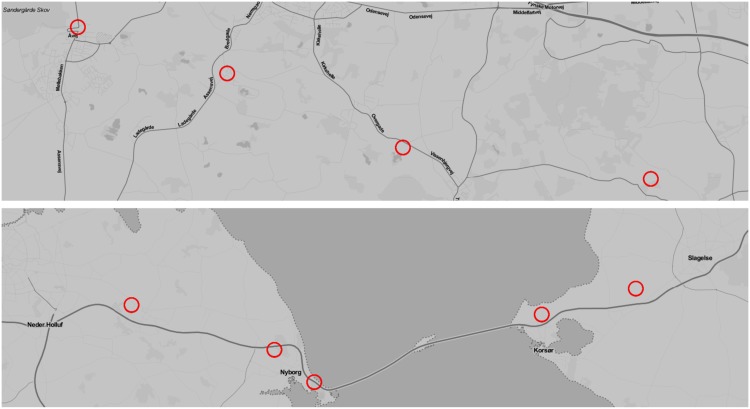
Two examples of stop-locations found using WiFi data which are not geographically stationary. Each plot shows one stop location inferred from WiFi data, each circle shows a single GPS estimation associated with the location. The two stop-locations are most likely based on access points which are present in a train or a bus.

Finally, there is the matter of non-stationary stop-locations in WiFi data. When using WiFi to detect stop-locations, it is possible to observe stop-locations which are not spatially stationary—this is for example due to personal MiFi devices and access points located in for example busses and trains. Examples of such non-stationary stop-locations are shown in Fig 9. When evaluating the start and stop-times of stop-locations, such non-stationary stop-locations will affect the results of the WiFi-based methods negatively.

We realize that using the data from a single subject for our study is a limitation to the generalizability of the findings. Nevertheless, the particular individual reveals mobility pattern at least as complex as we would expect from a typical adult: she works at two separate venues, appears to have two home locations (places visited on weeknights), and visits different areas of the city.

**Future work**. To achieve better results in the evaluations, one could filter out mobile routers—either by manually picking out SSID’s or by detecting routers which appear in different geographical locations. The former requires location-specific knowledge as each city/country has a different naming scheme for the routers on public transportation. The latter involves coupling the WiFi information with GPS data; in this work we intended to show that detecting stop locations is possible with just the WiFi data.

Further, in the proposed methods, we are not explicitly modeling the temporal dimension of the problem. If two routers are often observed close in time, the physical distance between them is likely to be low. Using this temporal closeness might also enable the construction of hierarchical clusters based on WiFi, consequently ameliorating the problem of scale.
